# Lysozyme and *Bacillus subtilis* improve growth performance, nutrient utilization, physiological stress indices, intestinal integrity, and gene expression of heat-stressed broilers

**DOI:** 10.1038/s41598-025-23910-2

**Published:** 2025-11-12

**Authors:** Ahmed M. Elbaz, G. S. Ramadan, Ahmed Ateya, M. G. Sallam

**Affiliations:** 1 Animal and Poultry Nutrition Department, Desert Research Center, Cairo, Egypt; 2 Animal Production Department,, National Research Centre, Agricultural and Biology Research Institute, Cairo, Egypt; 3 Department of Development of Animal Wealth, Faculty of Veterinary Medicine, Mansoura University, Mansoura, Egypt

**Keywords:** Broiler performance, Lysozyme, *Bacillus subtilis*, Immunity, Gene expression, Animal behaviour, Animal physiology

## Abstract

Feed additives play a crucial role in enhancing the poultry’s overall performance, whether by meeting their needs or helping to overcome various challenges, especially during heat stress. This study examined the effects of adding lysozyme and *Bacillus subtilis* on growth indices, lipid profiles, immune responses, gut integrity, and gene expression in heat-stressed broilers. A total of 600 one-day-old (Ross 308) male chicks were randomly allocated into four treatment groups (six replicates) as follows: CON, a basal diet without feed additives; B.S, a basal diet supplemented with *B. subtilis* (500 mg/kg diet); LYS, a basal diet supplemented with lysozyme (150 mg/kg diet); and BSLY, a basal diet supplemented with both *B. subtilis* and lysozyme (500 mg/kg and 150 mg/kg diet, respectively). Chickens were exposed to a temperature of 33 °C for 4 h daily during the experimental period. Results indicated that broilers receiving a diet supplemented with BSLY showed higher body weight gain and lower feed conversion ratio than the control (*p* < 0.05). The carcass characteristics and nutrient digestibility of the BSLY group were also improved. Total cholesterol decreased in chickens fed BSLY and B.S, while low-density lipoprotein (LDL) levels decreased in the LYS group. compared to the control group; however, high-density lipoprotein (HDL) increased in the BSLY, B.S, and LYS groups. Additionally, the addition of BSLY and B.S enhanced the immune response by increasing the levels of immunoglobulin G (IgG), immunoglobulin M (IgM), and the relative weight of the Bursa of Fabricius. Furthermore, gut integrity improved via expanding the ileum villus height in chickens fed BSLY, B.S, and LYS , as well as the counts of *Bacillus* spp. and *Lactobacillus* in the broilers fed BSLY and B.S. Dietary BSLY, B.S, and LYS modulated gene expression, via up-regulation of insulin-like growth factor 1 (IGF1), superoxide dismutase (SOD), glutathione peroxidase (GPx), and interferon‐gamma (IFN-γ) gene expression compared to the control group, while the highest SOD and IGF1 genetic modification was in the group that received BSLY. In conclusion, adding *B. subtilis*-lysozyme mixture can have a positive impact on growth performance indices, immune response, gut health, and gene expression, thereby increasing heat stress resistance of broiler chickens.

## Introduction

Several environmental factors cause considerable loss in poultry production, including heat stress and diseases. A recent study indicated that heat stress increases the risk of disease occurrence, resulting in general performance deterioration^1^, it has been shown that heat stress leads to negative effects on intestinal colonization and integrity by pathogenic bacteria such as *Escherichia coli* and *Clostridium perfringens*^2,3^. Additionally, heat stress can increase intestinal permeability by increasing peripheral blood flow and reducing intestinal blood supply, as well as, hypoxia, and increased exposure to oxidative stress by overproduction of reactive oxygen species, resulting in cell damage in intestinal tissue^4,5^. During heat stress, an oxidative stress response is often associated with the disruption of the intercellular junctional (an important part of the intestinal barrier)^6^, which compromises tight junctions, leading to a leaky gut and metabolic dysfunction^7,8^.

The broiler’s intestinal microbiota interacts with dietary formation, the intestinal mucosa, and the host’s immune responses, therefore, the microbial content of the intestines depends on many factors, the most essential of which are stress and diet^9^. Therefore, many nutritionists use feed additives to reduce heat stress’s harmful effects, such as probiotics, herbals, organic acids, etc.^10,11^. Therefore, this study aimed to reveal the possibility of reducing the harmful effects of stress by adding lysozyme and *B. subtilis* to heat-stressed broiler chicken diets.

Lysozyme is an enzyme (protein) that has an antibacterial effect, particularly against gram-positive bacteria via catalyzing the hydrolysis of mucopolysaccharides (polymeric compounds) that comprise the bacteria’s cell wall^12–14^. In addition, it forms part of the innate immune system by enhancing macrophage activation, immunoglobulin A (IgA) secretion, and clearance of bacterial pathogens^12^, which helps strengthen the immune system and improve anti-inflammatory response. Lysozyme is available in egg whites, saliva, tears, sweat, and mucus^13,15^. From this, lysozyme acts to improve performance through increasing growth, feed efficiency, and metabolic profile, as well as, enhancing gastrointestinal health, and altering the gastrointestinal bacteria ecology^12,16^. Adding beneficial microbes as feed additives for broiler chickens is common to support the health of the digestive system and enhance nutrient absorption, innate immunity, antioxidant status, and bacterial infection resistance^17,18^, thus contributing to improving broiler growth performance^19^. *Bacillus subtilis* is a beneficial microbe that has been approved as an animal feed additive, especially to resist harsh changing environmental conditions^20^, as well as, regulate some nutrient production (such as amino acids and fatty acids)^21^; therefore, it can be considered a viable alternative to antibiotics. In addition, it has a role in modifying the microbial content of the intestine by increasing the abundance of beneficial *Lactobacillus* and *Bifidobacterium* in the gut by consuming free oxygen^22^.

Given the multiple benefits demonstrated by many previous reports of *B. subtilis* and lysozyme supplements, we believe that adding them to the diets of chickens exposed to heat stress may have a positive effect in mitigating the harmful effects of heat stress. Therefore, this study aimed to investigate the effects of adding *B. subtilis* and lysozyme on growth, nutrient digestibility, immunity, antioxidant capacity, gene expression, and intestinal status of heat-stressed broilers.

## Results

### Growth performance

The effect of *B. subtilis* and lysozyme supplementation on growth performance is shown in Table 1. Significant decreases in the FCR (*p* < 0.05) were observed in groups fed BSLY and B.S compared to LSY and control groups during the start stage (0-21d); however, BWG tended to increase in B.S, LYS, and BSLY groups over the control group. Broilers fed treatment groups (BSLY, LSY, and B.S) had higher BWG (*p* < 0.05) and lower FCR than those in the control group during the grower stage (22-35d). During the overall period, supplementing broiler diets with the B.S, LYS, and BSLY improved BWG and FCR (*p* < 0.05) compared to other groups. However, BWG tended to have an increase in broilers fed BSLY than the B.S and LYS groups. Additionally, the best FCR in the group was feeding on BSLY. The average feed intake (AFI) of broilers did not differ (*p* < 0.05) among treatment groups in all experimental stages.

**Table 1:** Productive performance in broiler-fed diets supplemented with *B. subtilis* and lysozyme at 35 days.

	CON	B.S	LYS	BSLY	SEM	*p*-value
0–21d						
BWG (g/b)	751^b^	802^a^	773^ab^	806^a^	5.061	0.001
AFI (g/b)	928	941	932	939	8.354	0.106
FCR	1.23^a^	1.17^c^	1.20^b^	1.16^c^	0.026	0.001
22–35d						
BWG (g/b)	1029^c^	1107^b^	1085^b^	1148^a^	6.225	0.001
AFI (g/b)	2578	2650	2645	2649	7.413	0.214
FCR	2.50^a^	2.39^b^	2.43^b^	2.30^c^	0.009	< 0.001
0–35d						
BWG (g/b)	1780^c^	1909^ab^	1858^b^	1954^a^	2.590	< 0.001
AFI (g/b)	3507	3592	3578	3589	5.254	0.127
FCR	1.96^a^	1.88^c^	1.92^b^	1.83^d^	0.071	< 0.001

^a-d^Each trait with different superscripts differ significantly at *p* < 0.05; BWG, body weight gain; AFI, average feed intake; FCR, feed conversion ratio; CON, a basal diet without feed additive as the control group; B.S, added *B. subtilis* in the basal diet; LYS, added lysozyme in the basal diet; BSLY, added *B. subtilis* and lysozyme mixture in the basal diet.

### Carcass traits

The effect of *B. subtilis* and lysozyme supplementation on carcass traits is shown in Table 2. At the end of the experimental period, the experimental additives showed a positive effect on carcass traits. Dressing percentage increased (*p* < 0.05) while the abdominal fat content decreased in the chickens that received the B.S and BSLY compared to the other groups (*p* < 0.05). Furthermore, the relative weight of the intestines increased in chickens that received B.S, LSY, and BSLY (*p* < 0.05) compared to the control group; however, the highest intestine weight was in chickens that received BSLY and B.S. Additionally, liver, thigh, and breast weights were not affected (*p* < 0.05) by experimental additives.

**Table 2:** Carcass traits (%) and Nutrients digestibility (%) in broiler-fed diets supplemented with *B. subtilis* and lysozyme at 35 days.

	CON	B.S	LYS	BSLY	SEM	*p*-value
Carcass traits						
Dressing	69.1^b^	71.8^a^	70.6^b^	72.3^a^	6.194	0.001
Breast	24.1	24.3	23.9	24.2	9.005	0.147
Thigh	15.7	15.6	15.7	15.9	7.121	0.315
Liver	2.06	2.11	2.04	2.07	0.005	0.922
Abdominal fat	3.16^a^	1.54^b^	2.94^a^	1.38^b^	0.061	0.014
Intestinal weight	5.47^c^	6.35^a^	6.01^b^	6.42^a^	0.705	< 0.001
Nutrients digestibility						
Dry matter	65.8^c^	67.2^a^	66.7^b^	67.4^a^	0.025	0.001
Crude protein	70.3^d^	73.9^b^	72.5^c^	75.6^a^	0.114	< 0.001
Ether extract	67.1^b^	67.7^ab^	68.4^a^	68.9^a^	0.057	0.020
NFE	52.6	51.9	52.3	52.7	0.038	0.074

^a-c^Each trait with different superscripts differ significantly at *p* < 0.05; NFE, nitrogen-free extract; CON, a basal diet without feed additive as the control group; B.S, added *B. subtilis* in the basal diet; LYS, added lysozyme in the basal diet; BSLY, added *B. subtilis* and lysozyme mixture in the basal diet.

### Nutrients digestibility

Table 2 shows the effect of experimental supplements on nutrient digestibility. Chickens fed B.S and BSLY showed increased DM digestibility compared to other groups (*p* < 0.05). CP digestibility increased in chickens fed supplements with BSLY, B.S, and LYS compared to the control group; however, CP digestibility was better (*p* < 0.05) in chickens fed BSLY. Likewise, EE digestibility tended to be increased in the groups fed BSLY and LYS supplements compared to the B.S and control groups. Nevertheless, NFE digestibility was not affected between the experimental groups(*p* < 0.05).

### Serum constituents

**Fig. 1:**
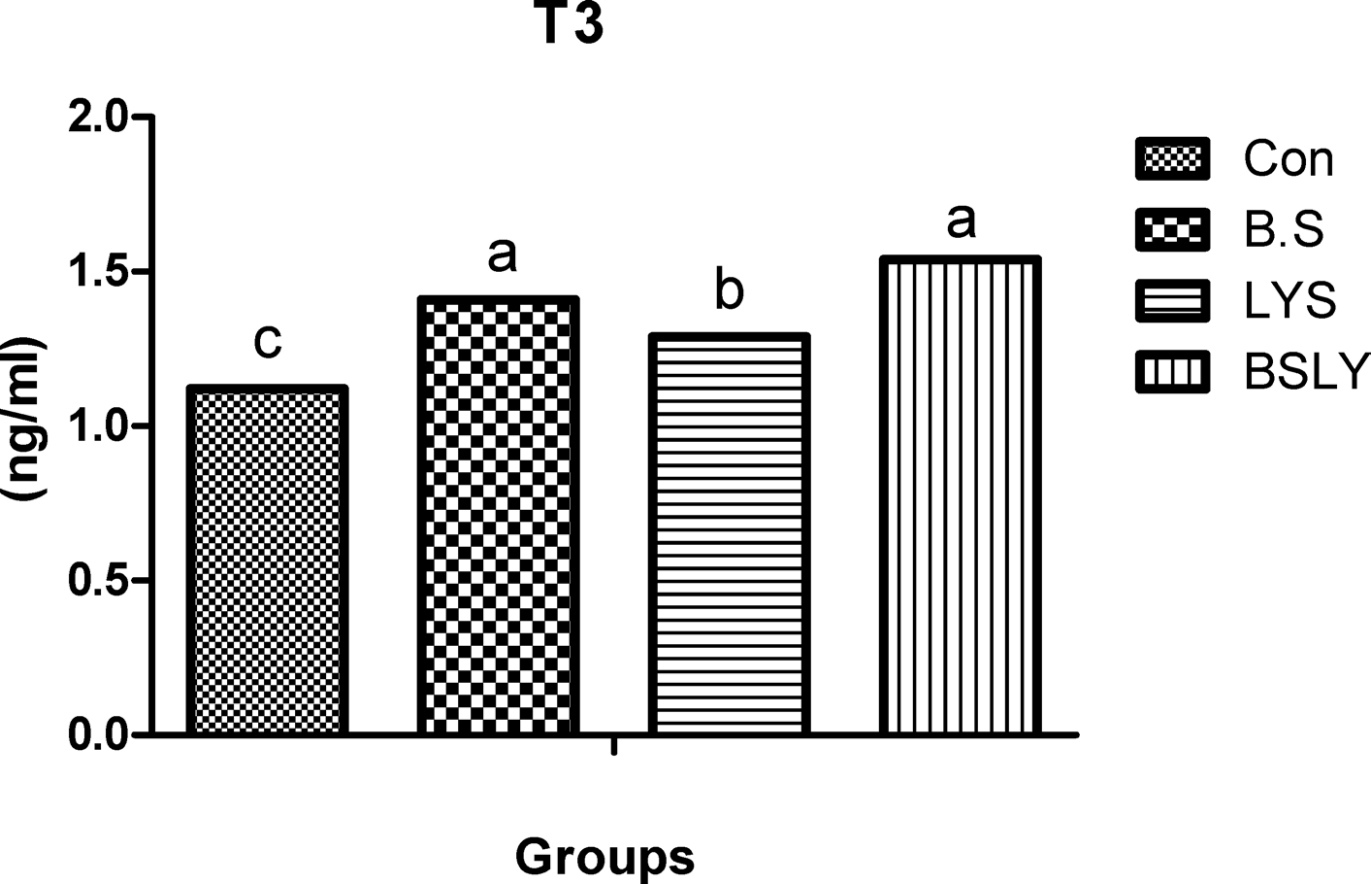
Effects of supplementation of *B. subtilis* and lysozyme on serum triiodothyronine (T3) hormone concentration of heat-stressed broilers. CON, a basal diet without feed additive as the control group; B.S, added *B. subtilis* in the basal diet; LYS, added lysozyme in the basal diet; BSLY, added *B. subtilis* and lysozyme mixture in the basal diet. Values with different superscript letters are significantly different (*p* < 0.05). Data are presented as the mean values with their standard errors.

**Fig. 2:**
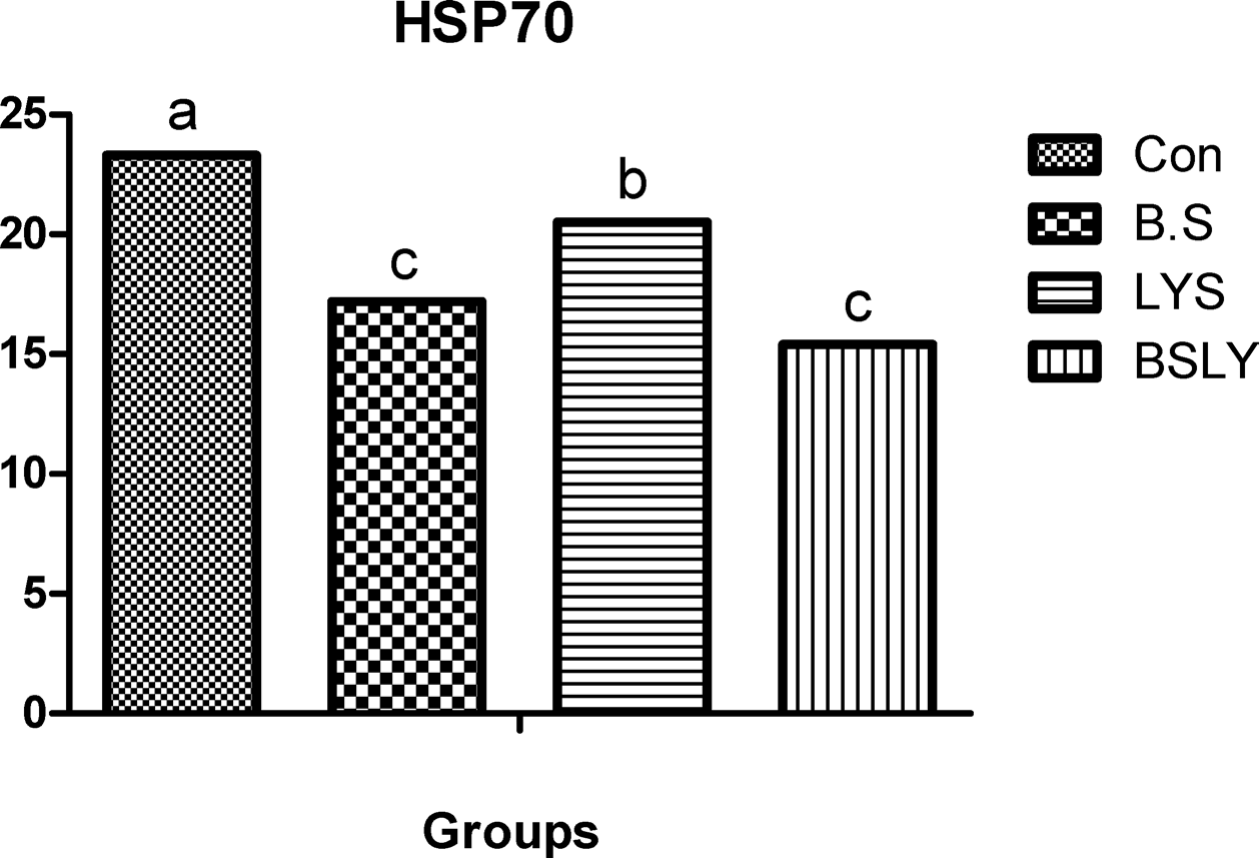
Effects of supplementation of *B. subtilis* and lysozyme on serum heat shock protein 70 (HSP70) concentration of heat-stressed broilers. CON, a basal diet without feed additive as the control group; B.S, added *B. subtilis* in the basal diet; LYS, added lysozyme in the basal diet; BSLY, added *B. subtilis* and lysozyme mixture in the basal diet. Values with different superscript letters are significantly different (*p* < 0.05). Data are presented as the mean values with their standard errors.

A significant change in blood lipid profiles, thyroid activity, and heat shock protein 70 (HSP70) by the experimental feed additives, as illustrated in Table 3 and Figs. 1 and 2. Serum HDL levels increased in chickens that received B.S., LYS, and BSLY, compared to the control group, while the highest HDL level was in the chickens fed BSLY. Cholesterol levels were lower (*p* < 0.05) in the groups receiving the B.S. and BSLY than in the LYS and control groups. Serum LDL levels tended to be decreased in chickens that received LYS and BSLY compared to the B.S and control groups. Nevertheless, the triglyceride level was not affected by the experimental treatments. Serum triiodothyronine (T3) hormone concentration increased (*p* < 0.05) in the broiler that received BSLY, LYS, and B.S compared to the control group; however, the highest increase (*p* < 0.05) was recorded in chickens receiving BSLY and B.S (Fig. 1). The experimental additives reduced (*p* < 0.05) the HSP70 in the serum compared to the control group, while the lowest level of HSP70 was in the chickens that received B.S. and BSLY compared to the other groups, as shown in Fig. 2.

**Table 3:** Serum lipid profile (mg /dl) in broiler-fed diets supplemented with *B. subtilis* and lysozyme at 35 days.

	CON	B.S	LYS	BSLY	SEM	*p*-value
Triglycerides	223	217	229	221	0.533	0.071
Cholesterol	216^a^	186^b^	209^a^	181^b^	0.271	0.004
LDL	123^a^	119^a^	104^b^	110^ab^	0.093	0.001
HDL	70.4^d^	76.8^b^	72.9^c^	78.4^a^	0.077	< 0.001

^a-c^Each trait with different superscripts differ significantly at *p *< 0.05; HDL, high-density lipoprotein; LDL, low-density lipoprotein; CON, a basal diet without feed additive as the control group; B.S, added *B. subtilis* in the basal diet; LYS, added lysozyme in the basal diet; BSLY, added *B. subtilis* and lysozyme mixture in the basal diet.

### Immune response

Experimental additives played a significant role in stimulating the immune response (immunoglobulins and lymphoid organs), as shown in Table 4. The IgG level increased (*p* < 0.05) in the chickens that received B.S, LYS, and BSLY than in the chickens that received the control diet. Likewise, the IgM level increased (*p* < 0.05) in the chickens that received B.S and BSLY than in the chickens that received the LYS and control diet, while IgA levels were unaffected by the experimental diet. The relative weight of the spleen and thymus were not affected among the experimental groups (*p* < 0.05), while, the relative weight of the bursa of Fabricius significantly increased in the groups that received B.S. and BSLY compared to the LYS and control groups.

**Table 4:** Immune response (immunoglobulins (mg/dl) and Lymphoid organs (%)) in broiler-fed diets supplemented with *B. subtilis* and lysozyme at 35 days.

	CON	B.S	LYS	BSLY	SEM	*p*-value
Immunoglobulins						
IgA	221	219	223	220	0.020	0.131
IgG	427^d^	575^b^	481^c^	688^a^	0.075	< 0.001
IgM	76.4^b^	91.8^a^	77.2^b^	89.7^a^	0.009	0.003
Lymphoid organs						
Spleen	0.37	0.36	0.37	0.38	0.097	0.085
Bursa of fabricius	0.15^b^	0.18^a^	0.16^b^	0.19^a^	0.055	0.001
Thymus	0.29	0.28	0.29	0.31	0.008	0.090

^a-d^Each trait with different superscripts differ significantly at *p* < 0.05; CON, a basal diet without feed additive as the control group; B.S, added *B. subtilis* in the basal diet; LYS, added lysozyme in the basal diet; BSLY, added *B. subtilis* and lysozyme mixture in the basal diet.

### Intestinal microbiota and morphology

The effect of supplementation of *B. subtilis* and lysozyme on the intestinal microbial and morphology (villus length (VH) and crypt depth (CD)) is shown in Table 5. The BSLY and B.S groups had the longest ileum VH, followed by the LYS and then control groups; moreover, VH: CD ratio increased (*p* < 0.05) in broiler fed BSLY, B.S, and LYS compared with the control group. Nevertheless, the chickens that received the BSLY and B.S had the highest VH and VH: CD ratio compared with the other groups. Moreover, crypt depth was not affected among the experimental groups compared to the control groups. Furthermore, the *Lactobacillus* counts increased (*p* < 0.05) in broiler fed BSLY compared to the LYS, B.S, and control groups. Likewise, *Bacillus spp.* counts increased (*p* < 0.05) in broiler fed B.S and BSLY compared to the LYS and control groups. Additionally, the count of *E. coli* decreased (*p* < 0.05) in BSLY, LYS, and B.S groups compared with the control groups.

**Table 5:** Cecum microflora (log10 CFU g^−1^) and ileum morphology in broiler-fed diets supplemented with *B. subtilis* and lysozyme at 35 days.

	CON	B.S	LYS	BSLY	SEM	*p*-value
Microflora count						
Bacillus spp.	4.53^b^	5.68^a^	4.87^b^	5.71^a^	0.117	0.001
Lactobacillus	5.84^c^	6.61^b^	6.27^bc^	7.03^a^	0.065	< 0.001
*Escherichia coli*	4.29^a^	3.56^b^	3.81^b^	3.23^c^	0.247	< 0.001
Morphology						
Villus height (μm, VH)	554^c^	761^ab^	712^b^	809^a^	11.84	0.001
Crypt depth (μm, CD)	78.0	80.2	81.3	80.7	0.741	0.064
VH: CD	7.10^c^	9.48^ab^	8.76^b^	10.0^a^	0.195	< 0.001

^a-c^Each trait with different superscripts differ significantly at *p* < 0.05; VH: CD, Villus height: Crypt depth; CON, a basal diet without feed additive as the control group; B.S, added *B. subtilis* in the basal diet; LYS, added lysozyme in the basal diet; BSLY, added *B. subtilis* and lysozyme mixture in the basal diet.

### Gene expression

**Fig. 3:**
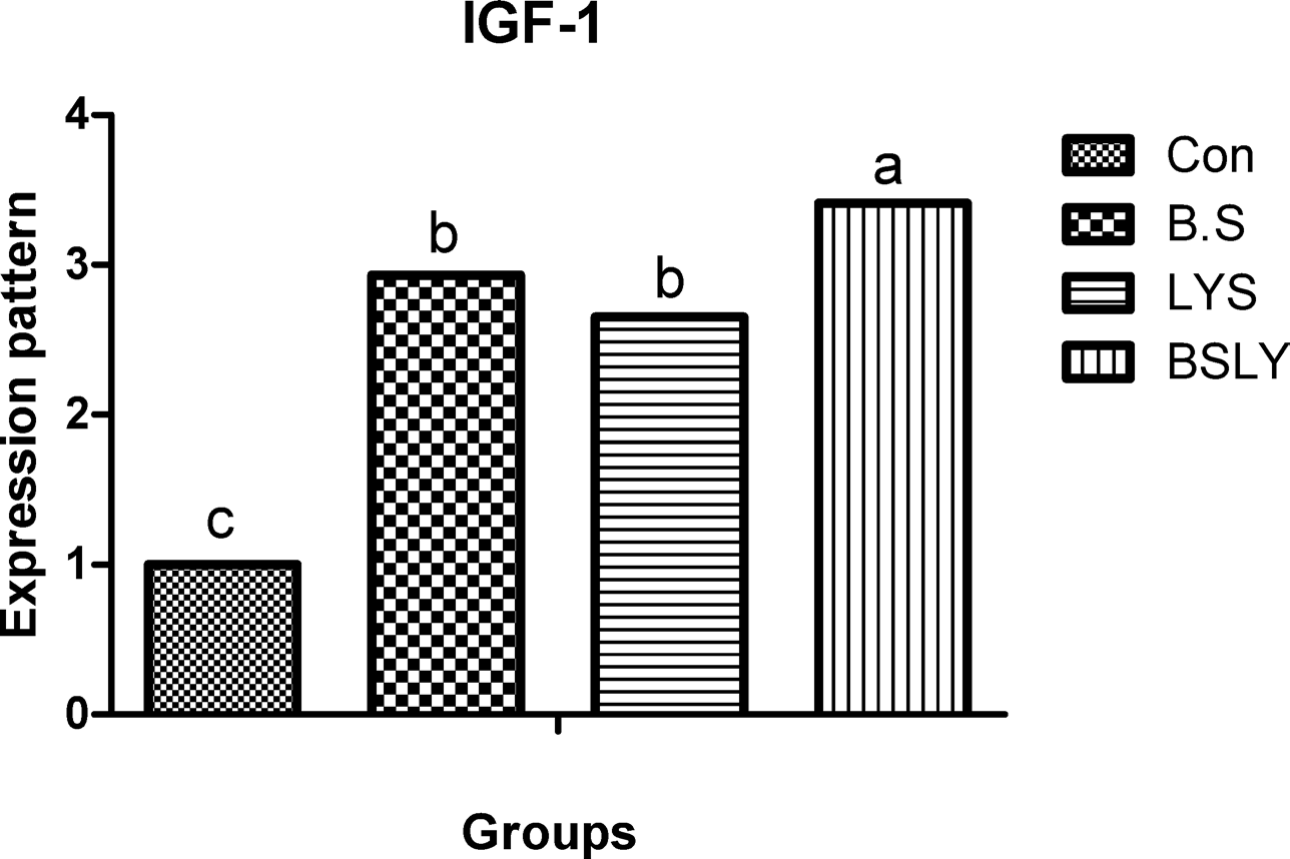
Effects of supplementation of *B. subtilis* and lysozyme on insulin-like growth factor 1 (IGF1) gene expression of heat-stressed broilers. CON, a basal diet without feed additive as the control group; B.S, added *B. subtilis* in the basal diet; LYS, added lysozyme in the basal diet; BSLY, added *B. subtilis* and lysozyme mixture in the basal diet. Values with different superscript letters are significantly different (*p* < 0.05). Data are presented as the mean values with their standard errors.

**Fig. 4:**
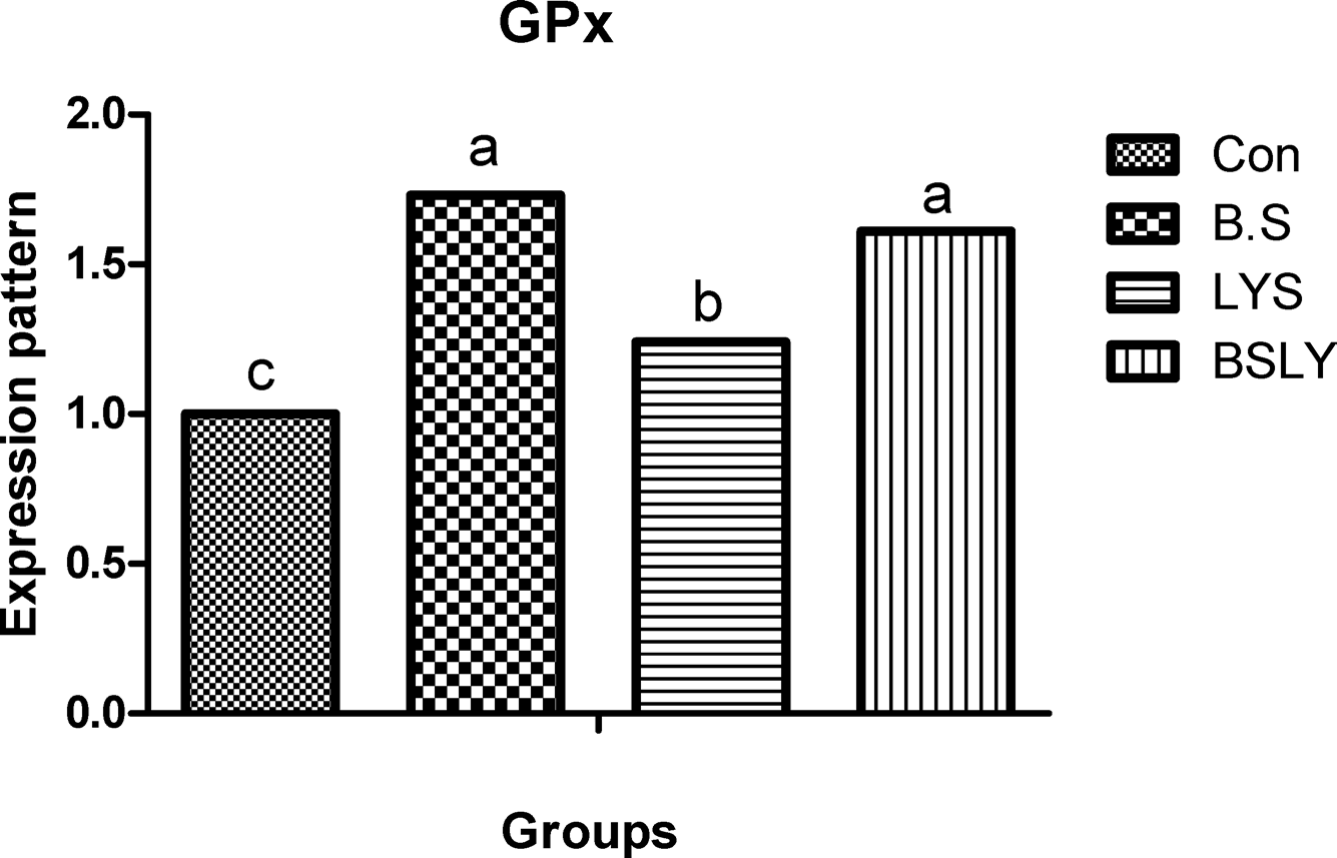
Effects of supplementation of *B. subtilis* and lysozyme on glutathione peroxidase (GPx) gene expression of heat-stressed broilers. CON, a basal diet without feed additive as the control group; B.S, added *B. subtilis* in the basal diet; LYS, added lysozyme in the basal diet; BSLY, added *B. subtilis* and lysozyme mixture in the basal diet. Values with different superscript letters are significantly different (*p* < 0.05). Data are presented as the mean values with their standard errors.

**Fig. 5:**
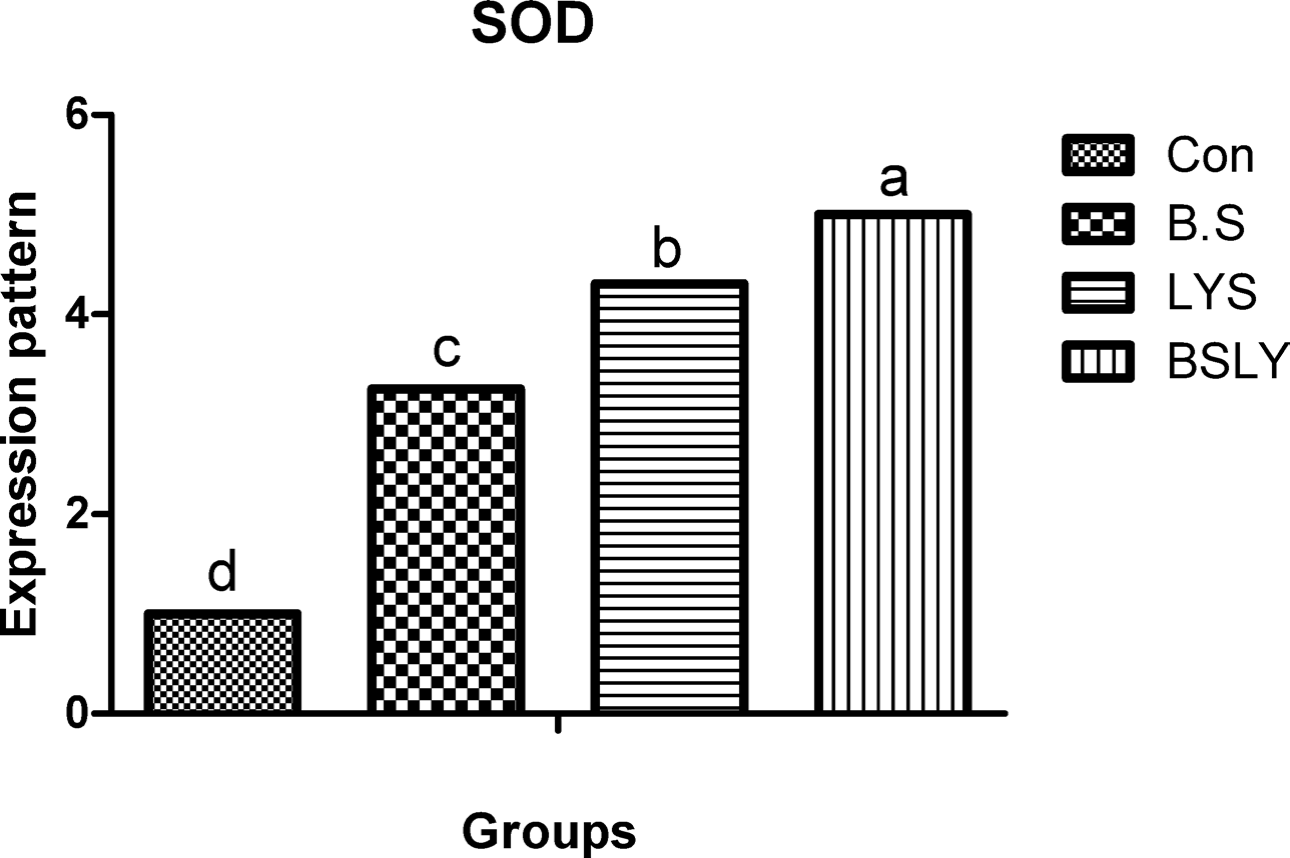
Effects of supplementation of *B. subtilis* and lysozyme on superoxide dismutase (SOD) gene expression of heat-stressed broilers. CON, a basal diet without feed additive as the control group; B.S, added *B. subtilis* in the basal diet; LYS, added lysozyme in the basal diet; BSLY, added *B. subtilis* and lysozyme mixture in the basal diet. Values with different superscript letters are significantly different (*p* < 0.05). Data are presented as the mean values with their standard errors.

**Fig. 6:**
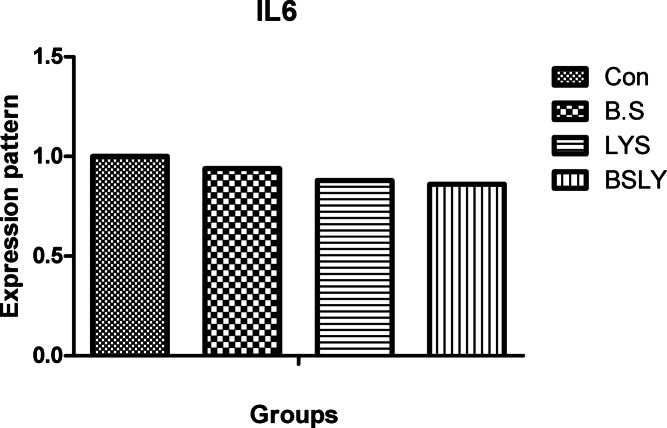
Effects of supplementation of *B. subtilis* and lysozyme on interleukin-6 (IL-6) gene expression of heat-stressed broilers. CON, a basal diet without feed additive as the control group; B.S, added *B. subtilis* in the basal diet; LYS, added lysozyme in the basal diet; BSLY, added *B. subtilis* and lysozyme mixture in the basal diet. Values with different superscript letters are significantly different (*p* < 0.05). Data are presented as the mean values with their standard errors.

**Fig. 7:**
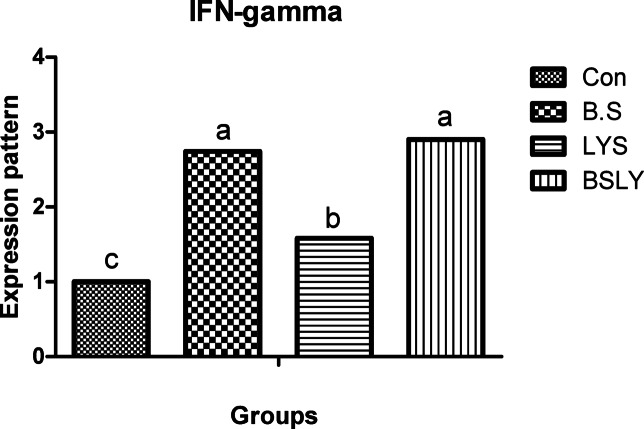
Effects of supplementation of *B. subtilis* and lysozyme on interferon‐gamma (IFN-γ) gene expression of heat-stressed broilers. CON, a basal diet without feed additive as the control group; B.S, added *B. subtilis* in the basal diet; LYS, added lysozyme in the basal diet; BSLY, added *B. subtilis* and lysozyme mixture in the basal diet. Values with different superscript letters are significantly different (*p* < 0.05). Data are presented as the mean values with their standard errors.

In the liver and spleen samples, mRNA expression of the IGF-I, GPx, SOD, and IFN-γ was significantly increased (*p* < 0.05) in broilers fed experimental supplements compared to control (Figs. 3,4,5,6 and 7); whereas, mRNA expression of IL-6 was not affected (*p* < 0.05) among the experimental groups. Gene expression of IGF-I and SOD was significantly increased (*p* < 0.05) in broilers fed BSLY, B.S, and LYS compared to the control group; while the highest IGF-I and SOD gene expression was in the BSLY group. Additionally, gene expression of GPx and INF-γ was significantly increased (*p* < 0.05) in BSLY, B.S, and LYS groups compared to the control group; while the highest GPx and INF-γ gene expression was in the B.S and BSLY groups.

## Discussion

Many previous studies have shown that heat stress causes a major threat to the poultry industry via disruption of intestinal structure and increases the risk of oxidative stress exposure, furthermore, poor feed utilization, resulting in deterioration of productive performance and increased mortality^1,17^. Many reports have proven the positive role of feed additives in alleviating the harmful effects of heat stress on birds^11,23,24^. As we expected, our results showed that the dietary addition of *B. subtilis* and lysozyme enhanced growth performance, nutrient utilization, immune response, and intestinal integrity, as well as, modified gene expression in heat-stressed broilers.

The current study showed a noticeable improvement in the productive performance of heat-stressed broiler chickens fed BSLY via increasing BWG and enhancing FCR. These results are consistent with El-Deep et al.^25^; Attia et al.^24^; and Elbaz et al.^9^ who reported that including lysozyme or probiotics as a dietary supplement improved BWG and FCR in monogastric animals. The findings are consistent with those of Abu Hafsa et al.^26^, who found that supplement lysozyme has an efficient impact in improving growing rabbit performance. Similar studies on chickens^27^, and rabbits^28^ observed that adding lysozyme increased growth while enhancing the intestinal microbes and gut barrier function, resulting in high digestion and absorption capacity. Furthermore, probiotics also play an important role in subsiding oxidative damage caused by heat stress in poultry^29^, by boosting intestinal development and enhancing produces digestive enzymes, and immune responses in chickens^1,23^. Additionally, the antimicrobial, and antioxidant properties of lysozyme^24,30^, resulting in enhanced intestinal integrity and feed utilization, thus enhancing growth performance. The improved growth performance of chickens fed *B. subtilis-* lysozyme mixtures may be due to the synergistic effect of *B. subtilis* and lysozyme, which increases protection against gut-harmful microorganisms and reduces oxidative stress, thus enhancing feed utilization of broiler chickens exposed to heat stress.

Deterioration of carcass characteristics is one of the most important losses incurred by the poultry industry during heat stress^1^. The results of our study compared to the control group, observed a noticeable improvement in the carcass characteristics of chickens that received the mixture of *B. subtilis* and lysozyme, through the dressing percentage and relative weight of intestines increased, while abdominal fat decreased. Results of the current study were supported by many studies, which indicate that improving the carcass weight of broilers^31^, and rabbits^26^ fed a diet containing lysozyme or probiotics. Consistent with the results by Tang et al.^31^ who reported that adding *B. subtilis* significantly increased the breast muscle weight and decreased abdominal fat of broilers. Carcass weight is a proportional measurement of the live body weight of a bird^24^, this is consistent with our results, as increased carcass weight corresponded with the improvement in BWG in broiler fed *B. subtilis-* lysozyme mixture and *B. subtilis* alone. The improvement in carcass weight in the current study may be due to the dual properties of both *B. subtilis* and lysozyme, the most important of which is antimicrobial, which enhances gut health (modification of microbial content) and nutrient utilization by increased digestion and absorption of nutrients, thus enhancing carcass characteristics.

Fat deposition in the carcass is associated with meat quality, feed efficiency, and economic gains for the poultry industry. Many reports have confirmed that reducing abdominal fat is an economic gain for slaughterhouses, as belly fat is considered a waste of slaughterhouses (dressing percentage is inversely related to the weight of abdominal fat)^32^. Our results showed a decrease in abdominal fat in chickens that received *B. subtilis* alone or combined with lysozyme compared to the control. Similar to the results of this study, Bidura et al.^33^ and Elbaz et al.^23^ noticed that the abdominal fat content in broilers that received probiotics was significantly lower than in the control group. Abdominal fat decreased in the groups fed *B. subtilis* may be reverted to the role of direct-fed beneficial microbes in the conversion of excess energy from the metabolism process or by the rate of fatty acid synthesis being reduced through a decrease in the activity of acetyl-CoA carboxylase (responsible enzyme limiting in fatty acid synthesis), by Santoso et al.^34^. Our results confirm the effect of combining *B. subtilis* and lysozyme or *B. subtilis* alone on regulating the distribution of carcass fat, which has a significant economic role in addition to enhancing meat quality.

The health of the digestive system is important for digesting and absorbing nutrients, in addition to maintaining animal health and performance^35^. The digestive system contains many diverse enzymes and microbes that contribute to digestion and the breakdown of complex nutrients into simple forms that can be easily digested and absorbed. Heat stress adversely affects enzymatic activity, and alters the diverse community of microorganisms, and the morphological structure of the small intestine, thus deteriorating general performance^1,36^. This is what the results of the current study showed, as nutrient utilization decreased in birds fed a basal diet without additives under heat stress. In addition, there was a significant enhancement in nutrient digestibility in the group that received a mixture of *B. subtilis* and lysozyme than other groups. Similar to the results of this study, Abu Hafsa et al.^26^ noticed that the digestibility coefficient of DM, CP, EE, and NDF improved in rabbits fed lysozyme-supplemented diets. The results of our study were supported by many studies, which indicate that enhancing the nutrient digestibility of broilers^23,31^, growing pigs^37^, and rabbits^28^ fed a diet containing lysozyme or probiotics. Furthermore, the probiotics supplementation improved growth performance, FCR, and intestinal absorptivity in broilers^38^. The observed improvement in nutrient digestion in the current study may be due to the antimicrobial and enzymatic activity-stimulating properties of the *B. subtilis*-lysozyme mixture, which reflects positively on the efficiency of feed utilization and the chicken’s performance.

The results of the current study demonstrated positive effects of lysozyme and *B. subtilis* supplementation on lipid profiles. Serum cholesterol and LDL levels decreased, while HDL levels increased in broiler fed lysozyme and probiotic supplementation mixture, this is consistent with many previous results^39,40^. The findings are consistent with those of Abu Hafsa et al.^26^, who found that supplement lysozyme has an important impact on lipids metabolism by reducing blood cholesterol, triglycerides, and LDL and increasing phospholipids and HDL levels. The effect of adding lysozyme on blood lipid profiles may be due to its role in affecting phospholipids, which play an important role in transporting excess cholesterol and triglycerides from the body to the liver^26^. The capacity of phospholipids to enhance biliary cholesterol excretion, reduce intestinal cholesterol absorption, and modify the enzymes involved in lipoprotein metabolism could explain these positive effects of supplement lysozyme on serum lipid profile^41^. Furthermore, the hypocholesterolemic influence of these supplements can be explained via their ability to deconjugate and dehydroxylate bile acids resulting in inhibiting fatty acid synthesis, lipids absorption, and increasing fatty acids fecal excretion, which leads to lowering lipid profiles in blood^10,39^. We conclude, that adding *B. subtilis* and lysozyme reduced serum lipid profile compared to the control group, indicating that dietary experimental supplements had a beneficial effect on lipid reduction in broilers under heat stress.

Detecting physiological changes (physiological stress indicators) due to stress is necessary to clarify the role of nutritional supplements in improving the physiological and health status of heat-stressed birds, including heat shock proteins (HSP70) and the activity of thyroid hormones (T3). HSP70 is one type of heat shock protein that is promptly synthesized in response to the bird being exposed to stressors (early response systems) such as feed restriction, and elevated temperature^42,43^. Heat stress is majorly responsible for the induction of reactive oxygen metabolites (ROM), HSP90, and HSP70^44^. Thus, the expression of HSP has been studied extensively and used as a marker for exposure to heat stress in broilers. The HSP70 gene plays an important role in protecting the body from the deleterious effects of oxidative stress in chickens when they are exposed to thermal conditions^42^, in which HSP70 expression in peripheral leukocytes, resulting in cellular defense mechanisms are activated by proinflammatory cytokine induction. Previous research has reported that HSP70 is involved in thermal acclimation and survival during exposure to heat stress^45^. In this study, serum HSP70 levels elevated significantly in broilers exposed to heat stress in the control group compared to broilers fed *B. subtilis* and lysozyme. The results of our study are consistent with other studies, as dietary supplements reduced the level of heat shock proteins (HSP60 and HSP70) in the blood and regulated gene expression in heat-stressed chickens^44,46^. In addition to its role in reducing cellular damage from heat stress and maintaining cellular homeostasis^47^. Antioxidants decrease the process of oxidation, thus decreasing the expression level of HSP70 in birds^48^. Hence it is suggestive that the administration of *B. subtilis* and lysozyme mixture was effective in decreasing the accumulation effect of heat stress as expected, this is due to the potent effect of feed additives used as effective antioxidants. Furthermore, the results of our study showed a significant increase in the level of the thyroid hormone (T3) in chickens fed *B. subtilis* and lysozyme compared to the control group. The current findings are consistent with those of Chotinsky and Mihaylov^49^ who found that supplementing *Lactobacillus* increased serum T3 concentration in broiler chickens. The thyroid gland is an endocrine organ that is involved in energy production by increasing the metabolic rate, in addition to being important in controlling metabolic rate^50^. Hence, *B. subtilis* and the lysozyme mixture addition is more effective in improving the nutrition metabolic rate by increasing blood T3 levels, which results in improved growth performance.

Many studies have shown the effects of heat stress on the immunosuppression of poultry^10,51^. During heat stress, intraepithelial lymphocytes, lymphoid organs, and IgA-secreting cells are negatively affected^35,52^. However, feed additives had an important positive role in enhancing the immune and functional response of birds exposed to heat stress^1,53,54^. This was confirmed by the results of the current study, where the level of IgG and IgM increased, in addition to the increase in the relative weight of the Bursa of Fabricius in a broiler fed mixture of lysozyme and *B. subtilis*. Similar to the results of this study, Qiu et al.^18^ noticed that the immune response in broilers that received probiotics was significantly enhanced than in the control group. The current findings are consistent with those of Chen et al.^55^ who found that supplementing lysozyme enhanced immune response in broilers. The antibacterial properties of dietary lysozyme suppressed the proliferation of pathogenic bacteria, promoting the response of the gut-associated immune system and improving rabbit^28^ and pig health^56^. The relative weight of a bird’s immune organs is an indicator of the bird’s immune status, as there is a direct correlation between the weight of the immune organ and the immune response^30^. Moreover, the increase in the level of immunoglobulins is associated with the relative weight of the immune organs^23^. The improvement in immune response may be due to the properties of antimicrobial, antioxidant, and anti-inflammatory in both *B. subtilis* and lysozyme that help in providing nutrients necessary for the development and increase of the immune organs by promoting the proliferation of lymphocytes and improving intestinal health^57^, resulting in stimulating the production of antibodies like immunoglobulin. In addition, JimeÂnez-Saiz et al.^58^ found that lysozyme hydrolyzed in the intestinal could be accompanied by the production of antimicrobial peptides, which may play efficacy roles in innate immunity. Our result concluded that the supplementation of *B. subtilis* and lysozyme has a positive influence on immunomodulation in broilers by providing more nutrients needed to develop the immune organs and synthesize antibodies.

Cecal microorganisms of broilers play an important role in nutrient utilization and immunity, amino acid, and vitamin production, and the products are fermented to generate short-chain fatty acids of nutritional value^59,60^, thus enhancing growth performance. Cecal microbiota is impacted by many factors, including season (heat stress), diet, and bird behavior^61^. Therefore, many attempts have been made to manipulate the microbiota cecal community, including strategies for changing the diet or adding feed-additive substrates (prebiotic and probiotic) to improve chicken growth rates by enabling the beneficial populations in the intestinal tract. This is what was shown by the results of the current study, where the addition of *B. subtilis* and lysozyme led to an increase in the beneficial microbes content (*Bacillus spp.* and *Lactobacillus*) and reduced harmful microbes content (*E. coli*) in the cecum compared to the control group. The current findings are consistent with those of Gong et al.^14^, Abdel-Latif et al.^27^, and Xia et al.^13^ who found that supplementing lysozyme enhanced microbial content by decreasing *E. coli* in broiler chickens. Similar to our results by Gao et al.^61^, and Jacquier et al.^62^ showed a decrease in *E. coli* and an increase in *Lactobacillus* in cecum when the addition of *B. subtilis* to broiler diets. *B. subtilis* exhibits both direct and indirect biocontrol mechanisms to suppress pathogens in broiler chickens, resulting in a more balanced gut microbiota, enhanced immune defenses, and a reduced risk of disease^19,63^. Adding *B. subtilis* reduces pathogenic bacteria through several mechanisms, including producing antibacterial metabolites, competitive colonization of the gut, and increasing beneficial bacteria through competitive exclusion and lactic acid production^19,64^. Additionally, lysozyme may support gut health by promoting beneficial bacteria and inhibiting harmful bacteria by destroying the cell walls of Gram-positive (harmful) bacteria^16,65^. This weakens the bacterial cell wall and makes it more susceptible to osmotic stress, which weakens its integrity and leads to dissolution and death. However, it may also benefit Gram-negative bacteria by modifying their cell walls^66^. Our results confirm the antimicrobial properties of combining *B. subtilis* and lysozyme by improving the host’s gut microbial balance and creating a favorable intestinal environment to support beneficial microbes in heat-stressed broilers.

The most important factors that need to be estimated to determine the carrying capacity of the intestine are studying CD, VH, VH: CD ratio, and the maintenance of intestinal mucosal integrity^25,67^, which is reflected on the bird performance. In the current study, VH, CD, and VH: CD ratio were measured as an indicator of intestinal integrity. Our results showed that the supplementation of *B. subtilis* and lysozyme had beneficial effects on gut histomorphology in broilers via the VH significantly increased. Similarly, He et al.^68^ found that implying dietary *Bacillus licheniformis* increases VH. These results are in agreement with several studies^62,69^. In this study, noticeable improvement in ileum histology may be due to the positive modification of the cecum microbiota as a result of supplementing *B. subtilis* and lysozyme, by reduces the pathogens, leads to cell integrity, and is attributed to the developing proliferation of crypt cells^63,66^. Additionally, they play a role in enhancing intestinal immunity and epithelial barrier integrity, which might improve the absorptive surface of the small intestine^69,70^. The increased VH in broilers fed *B. subtilis* and lysozyme mixture increases the surface area facing the digested nutrient that facilitates and increases absorption, resulting in improved growth performance in the broiler during heat stress.

To evaluate heat stress impacts different parameters have been used, including blood metabolites, and changes in microbial content, in addition, the most recent advances have explored expression profiling of genes that may play vital roles during heat stress. Different defensive activities are stimulated to protect the cells of tissues during stress, including the expression of stress response genes coding, like growth genes (IGF-I), antioxidant enzyme genes (SOD, and GPx), and immune-related genes such as proinflammatory (IL-6, and IFN-γ). Thus, the regulation of relevant genes under heat stress can act as a marker to identify the severity of stress. Heat stress in poultry leads to suppression of the immune system by regulating the expression of genes such as cytokines regulation, which are important markers of immune regulation, including interleukins (IL10 and IL-6), growth factors (IGF-I), tumor necrosis factors (TNFs), interferons (IFNs), and small peptide chemokines. During heat stress expression of proinflammatory cytokines is enhanced to activate immune functions via an enhanced proliferation of macrophages and lymphocytes^71^. Interestingly, in the current study, heat stress led to IGF1 decreased liver responsiveness and impact on the antioxidant system via repressing SOD and GPx genes which have major roles in inhibiting scavenging reactive oxygen species (ROS) generation in cells. Moreover, compared with the control group, IGF1 gene expression in the experimental group fed *B. subtilis* and lysozyme mixture increased significantly. In agreement with our results heat stress reduced protein deposition and reduced expression of the IGF1 gene in chicken breast^59^. Furthermore, Hassanien et al.^72^ observed a significant up-regulation of IGF expression in the liver of the probiotics-treated broiler groups. In addition, Liu et al.^73,74^, found that broiler fed *B. subtilis* increased the mRNA expression of pro-inflammatory cytokines in jejunal mucosa. Regarding the gene expression of antioxidant indicators, Bai et al.^75^ elicited that the expression of antioxidant enzyme genes, including SOD and GPx, was significantly improved when *B. subtilis* was added to the broiler diets. In the same respect, Abdel-Latif et al.^27^ reported that adding lysozymes to the broiler diet led to the mRNA expressions of Cu, Zn-superoxide dismutase (SOD1), glutathione peroxidase (GSH-Px), and interferon-gamma (IFN-γ) were up-regulated. The upregulation of gene expression of heat-stressed broilers indicates a protective role of the *B. subtilis* and lysozyme supplementation. Results of the study suggest that adding *B. subtilis* and lysozyme mixtures can have a significant impact on overall health by reducing stress and upregulation of the expression of proinflammatory cytokines via enhancing gut microbial diversity, subsequently improving intestinal barrier integrity and growth performance in heat-stressed broilers.

## Conclusions

Heat stress significantly impaired broiler performance parameters, as a result of poor vital functions and deterioration in gut integrity and oxidation defense system (modulates gene expression). However, supplementation of *B. subtilis* with lysozyme to the diet of broilers exposed to heat stress could not only ameliorate detrimental carcass changes and digestion and absorption of nutrients but also enhance immune responses and blood lipid oxidation–reduction compared to the broilers fed the control diet. Furthermore, combination of lysozyme and *B. subtilis* supports intestinal integrity by modifying morphology and microbial content. Moreover, dietary integration of *B. subtilis* and lysozyme modulates the expression of stress- and oxidation-related genes in broilers exposed to heat stress.

## Materials and methods

### Birds and treatments

Six hundred one-day-old male chicks (Ross 308) were purchased from a commercial hatchery (Wadi Poultry Company) and then divided into four experimental groups. Broiler chicks were weighed individually (40.9 ± 0.6 g) and completely randomly distributed among the experimental treatments in pens (30 birds/pen and 5 replicates/ treatment). The experimental treatments were as follows: CON, chicks fed a basal diet without feed additive; B.S, chicks fed a basal diet with the adding *B. subtilis* (500 mg/kg diet); LYS, chicks fed a basal diet with the adding lysozyme (150 mg/kg diet); BSLY, chicks fed a basal diet with the adding *B. subtilis* and lysozyme (500 mg/kg and 150 mg/kg diet, respectively). The nutritional program was based on two diets as follows; a starter diet (0 to 21 d, 3013 ME Kcal/Kg, and 23.08% protein) and a grower diet (21 to 35 d, 3109 ME Kcal/Kg, and 21.03% protein). Birds had ad libitum access to water and feed in mash form throughout experimental period. The experimental diets were formulated with corn and soybean meal to meet the nutrient specifications of broiler chicks based on NRC [^76^; Table 6], according to the Ross Catalog^77^. The temperature was regulated at 32.5 °C during the first three days, then gradually decreased to reach 21 °C at 35 days. The flock was exposed to a temperature of 33 °C starting on the fifth day for 4 h (beginning at 1 pm) throughout the experiment period. The lighting program was as follows: 24 h of light during the first five days, and 22 h of light from the sixth day until the end of the experiment. The lysozyme powder was provided by Zhejiang Aegis Biotech Co., Ltd, Hangzhou, China. *B. subtilis* strains (1.5 × 10^5^ CFU/g feed) were obtained from the Department of Microbiology, Faculty of Agriculture, Ain Shams University, Egypt.

**Table 6:** Experimental diet.

Feed ingredient %	Starter	Grower
Yellow corn	58.3	62.2
Soybean meal	32.6	27.6
Corn gluten meal	4.00	4.00
Dicalcium phosphate	1.80	2.00
Calcium bicarbonate	1.40	1.40
Methionine	0.20	0.20
Soya oil	1.20	2.10
Premix*	0.25	0.25
Salt	0.25	0.25
Chemical composition		
ME (kcal/kg)	3013	3109
Crude protein	23.08	21.03
Calcium	1.032	1.016
Available phosphorus	0.452	0.478
Lysine	1.150	1.053
Methionine	0.581	0.483

*Vitamin A 12,000 IU, vitamin D3 3000 IU, vitamin B12 0.02 mg, vitamin K3 3 mg, vitamin E 40 mg, vitamin B2 6 mg, vitamin B6 5 mg, vitamin B1 2 mg, biotin 0.075 mg, folic acid 2 mg, niacin 45 mg, pantothenic acid 12 mg, zinc 600 mg, copper 10 mg, iron 30 mg, manganese 100 mg, selenium 0.2 mg, iodine 1 mg, cobalt 0.1 mg.

### Productive performance and digestibility

Chickens and feed were weighed weekly by each pen and the mortality was recorded daily. Body weight gain (BWG) and average feed intake (AFI) were calculated to get the feed conversion ratio (FCR). On day 35, two birds per pen, totaling ten birds per experimental group were randomly selected, weighed, and slaughtered (euthanasia), chickens were fasted for 6 h before slaughter, for carcass index. The carcass, breast, thigh, liver, abdominal fat, and intestinal weight for each slaughtering chicken were calculated as a relative percentage of live body weight. Lymphoid organs such as the spleen, the bursa of Fabricius, and the thymus were weighed as an immune index.

On day 35, one chicken per replicate (5 birds/ group) was taken and placed in battery cages with a wire mesh bottom and excreta collection trays. Each cage has separate water and feed sources for each bird. The chickens were starved for 12 h before the start of the digestion experiment (to initialize). After that, excreta were collected for 4 days, with an average of every 12 h each day. The remaining feed and feathers in the excreta trays were carefully removed and weighed. Excreta were dried and stored in sealed bags at − 20 °C for analysis later. Excreta and feed samples were analyzed for dry matter (DM, # 934.01), ether extract (EE), nitrogen-free extract (NFE), and crude protein (CP, #990.03) using routine procedures AOAC^78^. The apparent digestibility coefficient is then calculated using the formula: ((Ingested nutrient (g) − Excreted nutrient (g))/Ingested nutrient (g)) × 100, according to Abreu et al.^79^.

### Serum biochemical analysis

On day 35, eight chickens from each group were selected blood samples were collected in tubes without anticoagulants were left to clot, and serum was collected by centrifuging (1500 × g for 15 min). Serum lipid profile concentrations were evaluated, including triglycerides, cholesterol, low-density lipoprotein (LDL), and high-density lipoprotein (HDL) using a spectrophotometer (Shimadzu UV 1601) by commercial kits (Boerne, Texas, USA) according to manufacturer protocols. Serum immunoglobulins, including IgM, IgA, and IgG levels, were determined using chicken-specific IgM, IgA, and IgG ELISA quantitation kits (Bethyl Laboratories Inc., Montgomery, TX, USA). Serum triiodothyronine (T3) hormone concentration was measured by radioimmunoassay with a kit which was produced by the Institute of Isotopes Co., Ltd. (Budapest, Hungary), and the samples were counted on Packard Gamma Counter (Perkin- Elmer Inc., Branford, CT, USA). Serum heat shock protein 70 (HSP70) concentration was measured by chicken HSP70 ELISA kits (QAYEE-BIO, Shanghai, China) based on the manufacturer’s instructions, as described by Humam et al.^80^.

### Intestinal microflora and morphology

At the end of the experiment, six birds from each group were sampled, for microbial enumeration in the cecum and histological measurements in the ileum, and 1 g of their cecum contents was serially diluted from 10^−1^ to 10^−10^ in saline solution, then, dilutions were plated onto nutrient agar (Merck, Germany), MRS agar (Merck, Germany), and MacConkey agar (Merck, Germany) and incubated for 48 h on 37 °C to specify *Bacillus spp*., *Lactobacillus spp*., and *Escherichia coli* counts, respectively. For morphology analysis, small segments (~ 3 cm) from the ileum sample were collected, and stored in a 10% formalin saline solution even analysis. Ileal slides (4–5 μm thickness) were cut using a rotary microtome (Thermo Shandon) and examined under a light microscope (E600; Nikon). Villus height (VH), crypt depth (CD), and VH: CD ratio were measured as described by Abdel-Moneim et al.^81^.

### Gene expression

On day 35 following the commencement of the trial (at the end of 5 weeks), 15 chickens from each group (selected at random as three chickens per replicate) were sacrificed by injection of pentobarbital sodium at 60 mg/kg live weight for sampling. Liver and spleen samples were taken and kept at − 80 °C until analysis. RNA was extracted from the liver and spleen using a QIAamp RNeasy Mini kit (Qiagen, Germany, GmbH). Relative expression of hepatic growth (IGF-I), antioxidant (SOD, and GPx), and pro-inflammatory (IL-6, and IFN-γ) indicators was assessed. The oligonucleotide sequence of the used primers (Metabion, Germany) is listed in Table 7. The reaction mixture was carried out in a Stratagene MX3005P real-time PCR machine with a total volume of 25 µl consisting of total RNA template 3 µl, 12.5 μl of 2 × QuantiTect Probe RT-PCR Master Mix, 8.125 μl PCR grade water, 0.5 μl of each primer of 20 pmol concentration and 0.125 μl of each probe (30 pmol), and 0.25 μl of QuantiTect RT Mix. At the end of the amplification phase, a melting curve analysis was performed to confirm the specificity of the PCR product. The relative expression of each gene per sample in comparison with ß. actin gene was carried out and calculated according to the 2^−ΔΔCt^^82^.

**Table 7:** Oligonucleotide sequence of the used primers in real time PCR.

Target gene	Oligonucleotide sequence (5′-3′)	Accession number
*IGF-1*	F: ATAGAGCCTGCGCAATGGAA	NM_001004384.3
	R: ACACAGTGTGCATCTTCACC	
*SOD*	F: AGGGGGTCATCCACTTCC	NM_205064.2
	R: CCCATTTGTGTTGTCTCCAA	
*GPX*	F: TTGTAAACATCAGGGGCAAA	NM_001163245.2
	R: ATGGGCCAAGATCTTTCTGTAA	
*IL-6*	F:GCTCGCCGGCTTCGA	NM_204628.2
	R:GGTAGGTCTGAAAGGCGAACAG	
*IFN-γ*	F: AAACAACCTTCCTGATGGCGT	NM_205149.2
	R:CTGGATTCTCAAGTCGTTCATCG	
*ß-Actin*	F: CCACCGCAAATGCTTCTAAAC	NM_205518.2
	R: AAGACTGCTGCTGACACCTTC	

### Statistical analyses

Data statistical measurements were handled with the SPSS programming tool (IBM SPSS. 201, Coppell, TX, USA) using One-way ANOVA followed by Tukey’s multiple range tests. All declarations of significance depended on *p* < 0.05.

## Data Availability

All data generated or analyzed during this study are included in this published article.

## Abbreviations

*AFI*: Average feed intake
*B. subtilis*: *Bacillus subtilis*
*B.S*: A basal diet with the addition of *B. subtilis*
*BSLY*: A basal diet with the addition of *B. subtilis* and lysozyme
*BWG*: Body weight gain
*CD*: Crypt depth
*CON*: A basal diet without feed additive
*CP*: Crude protein
*DM*: Dry matter
*EE*: Ether extract
*FCR*: Feed conversion ratio
*GPx*: Glutathione peroxidase
*HDL*: High-density lipoprotein
*IFN-γ*: Interferon‐gamma
*IgA*: Immunoglobulins A
*IGF*: Insulin-like growth factor 1
*IgG*: Immunoglobulins G
*IgM*: Immunoglobulins M
*IL-6*: Interleukins 6
*LDL*: Low-density lipoprotein
*LYS*: A basal diet with the addition of lysozyme
*NFE*: Nitrogen-free extract
*NRC*: National research council
*SOD*: Superoxide dismutase
*T3*: Triiodothyronine
*VH*: Villus height
